# Differential association of EphA2 intracellular regions in biased signaling

**DOI:** 10.1016/j.jbc.2025.108383

**Published:** 2025-03-04

**Authors:** Elmer Zapata-Mercado, Randall R. Rainwater, Ece Özdemir, Evgenia.V. Azarova, Mateusz A. Krzyscik, Elena B. Pasquale, Kalina Hristova

**Affiliations:** 1 Department of Materials Science and Engineering and Institute for NanoBioTechnology, Johns Hopkins University, Baltimore, Maryland, USA; 2 Sanford Burnham Prebys Medical Discovery Institute, La Jolla, California, USA

**Keywords:** receptor, signaling, fluorescence, FRET, bias

## Abstract

Biased signaling is the ability of a receptor to differentially activate certain signaling cascades in response to different ligands. Our previous work demonstrated that the monomeric ephrinA1 ligand and the widely used dimeric ephrinA1-Fc ligand induced EphA2 receptor tyrosine kinase (RTK)–biased signaling. The hypothesis that RTK biased signaling is a consequence of differential interactions between receptor intracellular regions when different ligands are bound to the extracellular region has not been experimentally verified thus far, in part because of the lack of high-resolution structures of full-length RTK oligomers. Here, we compare the effects of deletion of intracellular regions in EphA2 oligomers bound to the biased ligands, monomeric ephrinA1 or ephrinA1-Fc. Our data reveal distinct differences in the intracellular organization of EphA2 oligomers bound to the two ligands, supporting the hypothesis. They also suggest that EphA2 signaling could be modulated by agents that alter interactions between oligomerized EphA2 intracellular regions by binding at sites that can be distant from the ATP-binding pocket.

In response to extracellular ligands, cell surface receptors initiate downstream signaling cascades that control cell physiology. Some ligands can induce differential activation of receptor downstream signaling cascades, a phenomenon known as “biased signaling.” While most studies of biased signaling have thus far focused on G-protein–coupled receptors, there have also been accounts of receptor tyrosine kinase (RTK) biased signaling (1, 2, 3, 4). For instance, we have shown that the EphA2 RTK can engage in biased signaling (5). RTKs represent the second largest receptor superfamily and control cell growth, differentiation, motility, and survival (6, 7, 8).

EphA2 is highly expressed in epithelial and endothelial cells, where it triggers diverse downstream signaling pathways that regulate the assembly of cell–cell junctions (9). This receptor has been implicated in many physiological and disease processes, such as cancer (10, 11, 12), pathological angiogenesis (13, 14, 15, 16, 17), inflammation (13, 18, 19, 20), cataracts (21, 22, 23, 24), psoriasis (25), and parasite infections (11, 26). Like all RTKs, EphA2 is a single-pass transmembrane receptor with an extracellular region that binds the activating ligands (ephrins) and an intracellular region that contains the tyrosine kinase domain. The EphA2 intracellular region also contains a juxtamembrane segment and a sterile alpha motif (SAM) domain near its C terminus. SAM domains are α-helical cytoplasmic interaction modules that are only found in RTKs of the Eph receptor family.

EphA2 exists predominantly in monomeric and dimeric forms when not ligand-bound (27). In response to its ligands, the ephrinAs, EphA2 receptor molecules associate into large oligomers and crossphosphorylate each other on specific tyrosine residues (28). The EphA2 ligands, ephrinA1 through ephrinA5, are typically anchored to the surface of neighboring cells. However, they can also be released from the cell surface and activate EphA2 as soluble monomeric ligands. Recently, we showed that the soluble monomeric ephrinA1 (m-ephrinA1) can induce biased signaling when compared with the widely used ligand ephrinA1-Fc, which consists of the ephrinA1 extracellular region dimerized by fusion to the Fc region of an antibody (5). Two distinct responses were probed and compared: (i) phosphorylation of tyrosine 588 (Y588) in the juxtamembrane segment of EphA2, which represents one of the early events in EphA2 ligand–induced activation and (ii) inhibition of AKT S473 phosphorylation, which occurs independently of Y588 phosphorylation (29). A bias coefficient was calculated, which revealed that m-ephrinA1 exhibits a preference toward AKT inhibition compared with Y588 phosphorylation relative to ephrinA1-Fc (5). Fluorescence intensity fluctuation (FIF) spectrometry further showed that these two ligands stabilize EphA2 oligomers with different size distributions (27). However, no measurable correlation between the average EphA2 oligomer size and the magnitude of ligand bias was found when comparing average oligomer sizes (orders) and bias coefficients for m-ephrinA1, ephrinA1-Fc, and engineered peptide ligands (27).

We have shown that there are distinct differences in the organization of m-ephrinA1- and ephrinA1-Fc-bound EphA2 oligomers (30). This has been demonstrated through mutagenesis of two distinct extracellular interfaces that were first observed in crystal structures of isolated EphA2 extracellular oligomers (31). Both these interfaces are also engaged in full-length EphA2 oligomers stabilized by ephrinA1-Fc, but only one of them is engaged in the case of m-ephrinA1-bound oligomers, indicative of different arrangements of the m-ephrinA1 and ephrinA1-Fc-bound oligomers (30). However, it is not known if conformational differences also exist in the intracellular portion of EphA2 oligomers bound to the two ligands. It has been argued that the unstructured linkers between RTK domains prevent any allosteric effects, and thus structural information cannot be transmitted along the length of an RTK (32, 33, 34). Currently, there are no high-resolution structures of full-length RTK oligomers, which makes this hypothesis difficult to evaluate. Indeed, the intracellular regions of full-length RTKs have not yet been resolved in cryo-EM structures. Most likely, this is because the intracellular regions in the oligomers interact with each other dynamically through multiple interfaces, ensuring autophosphorylation on different tyrosines and interaction with different signaling effectors (35).

Here, we explore possible differences in the arrangement of EphA2 intracellular regions in EphA2 oligomers bound to m-ephrinA1 or ephrinA1-Fc, since differential interactions may contribute to differential phosphorylation of tyrosine residues in EphA2 and/or differential interaction with effector proteins (36). In the absence of high-resolution structural information, we sought to quantify the contributions of the entire intracellular region, or the SAM domain only, to the size and stability of EphA2 oligomers bound to m-ephrinA1 or ephrinA1-Fc. Toward this goal, we used FIF spectrometry and FRET to compare the lateral homoassociations of EphA2 WT and the two mutants lacking either the entire intracellular region or only the SAM domain. The observed differences in the contribution of the entire intracellular region or the SAM domain to the stability and structural arrangement of EphA2 oligomers are indicative of differences in the organization of the EphA2 intracellular portions bound to different ligands and are consistent with biased signaling.

## Results

### Bias plots demonstrate EphA2-biased signaling

**Figure 1:**
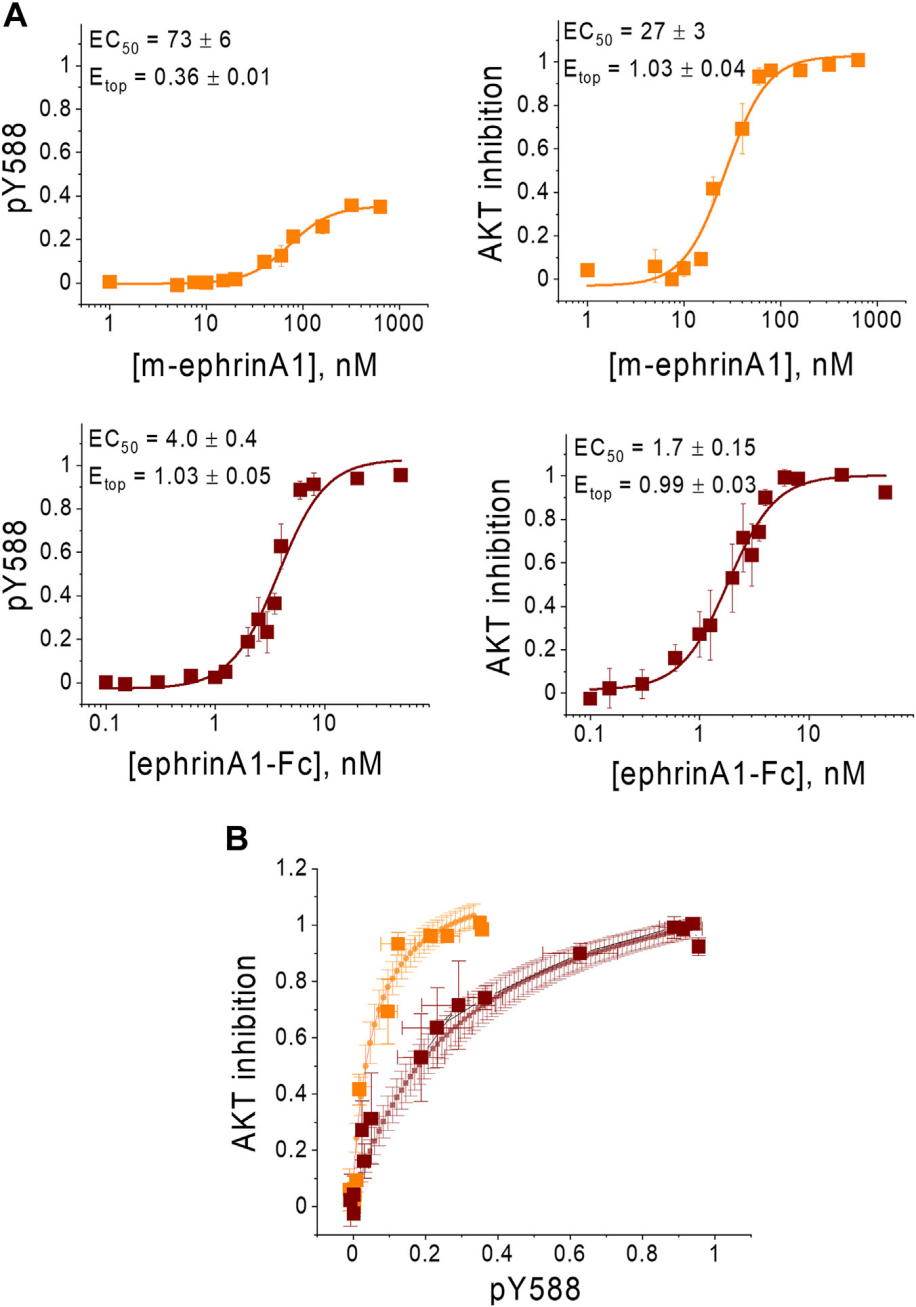
**A demonstration of EphA2 biased signaling.***A,* dose–response curves for EphA2 Y588 phosphorylation and AKT inhibition as a function of m-ephrinA1 and ephrinA1-Fc concentration. *B,* bias plot, where AKT inhibition levels are plotted *versus* Y588 phosphorylation (pY588) levels at the same ligand concentration. The theoretical line is given by Equation 2, where E_top_ and EC_50_ are the best-fit values for the dose–response curves in (*A*). Data are from Ref. (5), and theory was derived in Ref. (41). m-ephrinA1, monomeric ephrinA1; Y588, tyrosine 588.

In prior work, we measured dose–response curves for EphA2 Y588 phosphorylation and downstream AKT inactivation (decreased S473 phosphorylation) induced by the m-ephrinA1 and ephrinA1-Fc ligands (Fig. 1*A*). These dose–response curves can be fitted with the general Hill equation (Equation 1) to determine the efficacies and potencies of m-ephrinA1 and ephrinA1-Fc in inducing the two responses (5). A bias coefficient was calculated based on the efficacy and potency best-fit values and revealed that m-ephrinA1 exhibits preference toward AKT inhibition compared with Y588 phosphorylation relative to the reference ligand ephrinA1-Fc (5).

Such bias coefficients are widely used in cell signaling research, since they give information about the magnitude and direction of the bias and allow evaluation of statistical significance. However, a visual tool called “bias plot” is considered the most reliable tool to visualize the existence and direction of biased signaling (37, 38, 39). Here, for the first time, we construct a bias plot for Y588 phosphorylation and AKT inhibition, the two measured EphA2 responses induced by the ligands, m-ephrinA1 and ephrinA1-Fc.

The bias plot shows the magnitude of one response (y value in the dose–response curve) against the magnitude of the second response (for the same x value of the dose–response curve) for each ligand concentration (40). In the bias plot for Y588 phosphorylation and AKT inhibition induced by the ligands m-ephrinA1 and ephrinA1-Fc, the data points outline the bias trajectories for the two ligands (Fig. 1*B*). The solid lines represent the theoretical curves, given in Equation 2 (41), and the error bars denote the 68% confidence intervals. Bias plots are useful because the trajectories for the two ligands can be directly compared with each other (38). In Figure 1*B*, the m-ephrinA1 trajectory deviates toward the direction of the “AKT inhibition” axis and away from the “Y588 phosphorylation” axis compared with the trajectory of the ephrinA1-Fc reference ligand. The bias plot therefore shows that m-ephrinA1 exhibits bias toward AKT inhibition compared with Y588 phosphorylation relative to the reference ligand ephrinA1-Fc. This visual demonstration is in agreement with the bias coefficient calculations reported previously (5). Furthermore, the statistical significance of the difference between the two curves in Figure 1*B* can be evaluated by comparing the areas under the curve for the common range of x values (pY588 values from 0 to 0.36) (41). The difference between the two areas (0.27 ± 0.02 for m-ephrinA1 and 0.14 ± 0.02 for ephrinA1-Fc) is highly statistically significant (p < 0.0001) by Student’s *t* test. Thus, the existence of bias in EphA2 signaling in response to m-ephrinA1 and ephrinA1-Fc has now been demonstrated using two well-established methods: biased coefficients, as previously reported (5), and bias plots, as shown in Figure 1*B*. While these two types of analysis can sometimes yield different results (37), they are in agreement for EphA2. EphA2 can therefore be used as a prototype RTK to examine whether m-ephrinA1 and ephrinA1-Fc stabilize EphA2 oligomers in which the intracellular regions associate differently with each other, as could be expected for ligands inducing receptor-biased signaling (36).

### Distinct contribution of EphA2 intracellular regions to oligomerization induced by ligands that promote biased signaling

To assess how the EphA2 intracellular region affects oligomerization induced by the m-ephrinA1 and ephrinA1-Fc ligands, we used FIF, an imaging technique that calculates molecular brightness in small sections of the plasma membrane and yields a histogram of these molecular brightness values. The molecular brightness is calculated as the ratio of the variance in the fluorescence signal and the average fluorescence, after correcting for detector noise (Equation 3 and (42)). The magnitude of the molecular brightness is known to scale with the average oligomer size (42), and thus, the oligomerization state of a membrane protein can be revealed by comparison with a dimer control (TrkA [tropomyosin kinase receptor A] in the presence of saturating concentrations of its ligand nerve growth factor (27)). Larger brightness values than those of the dimer control indicate the presence of higher order oligomers.

**Figure 2:**
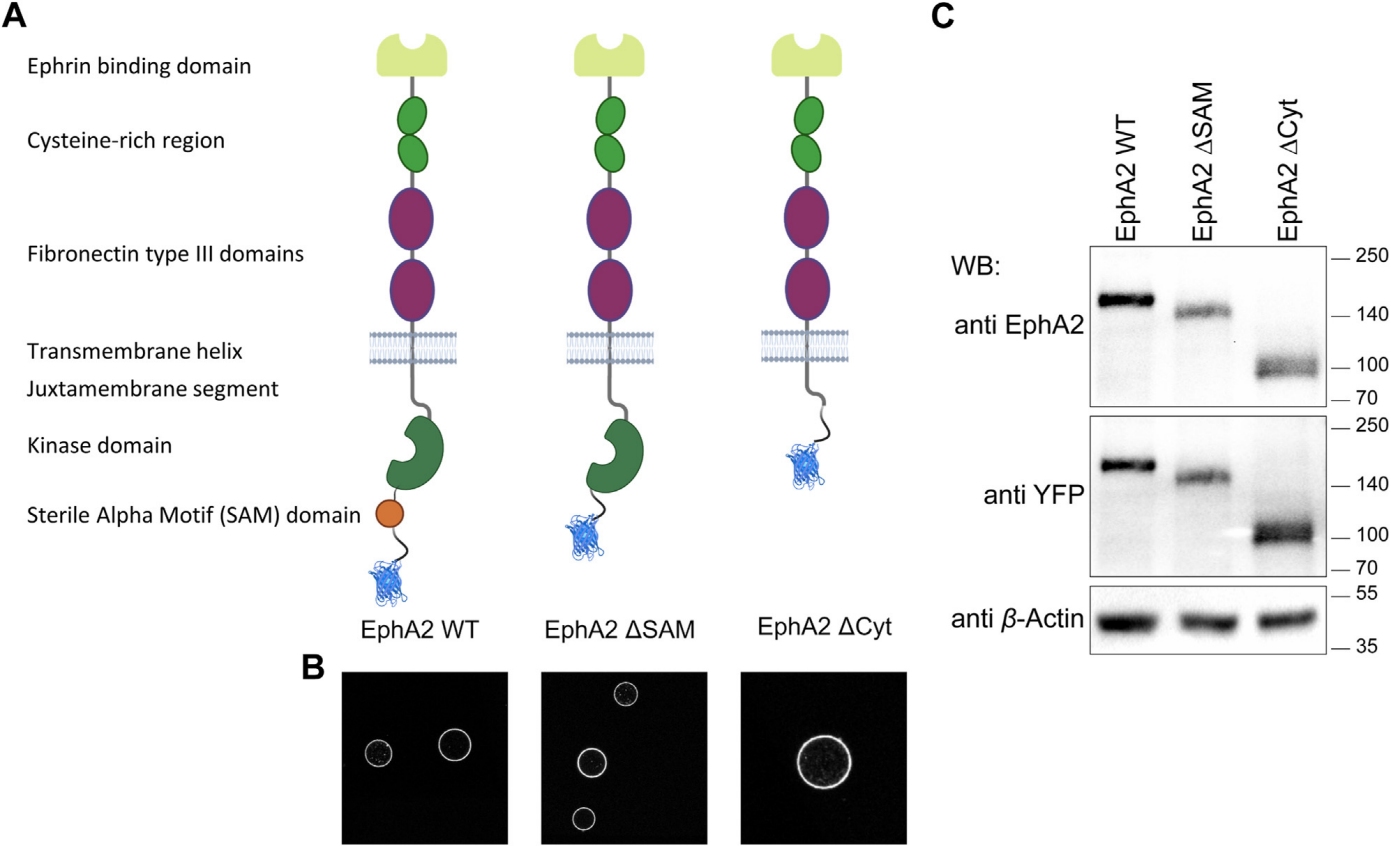
**Characterization of EphA2 ΔSAM and EphA2 ΔCyt truncation mutants.***A,* schematic representation of the EphA2 mutants used in this work. *B,* all constructs localize to the membrane of plasma membrane–derived vesicles, indicating that they are trafficked to the plasma membrane. *C,* Western blot performed with an anti-EphA2 antibody that recognizes the extracellular region of EphA2 and an anti-GFP antibody that recognizes the fluorescent protein (eYFP) fused to the C terminus of EphA2. β-Actin serves as a loading control.

**Figure 3:**
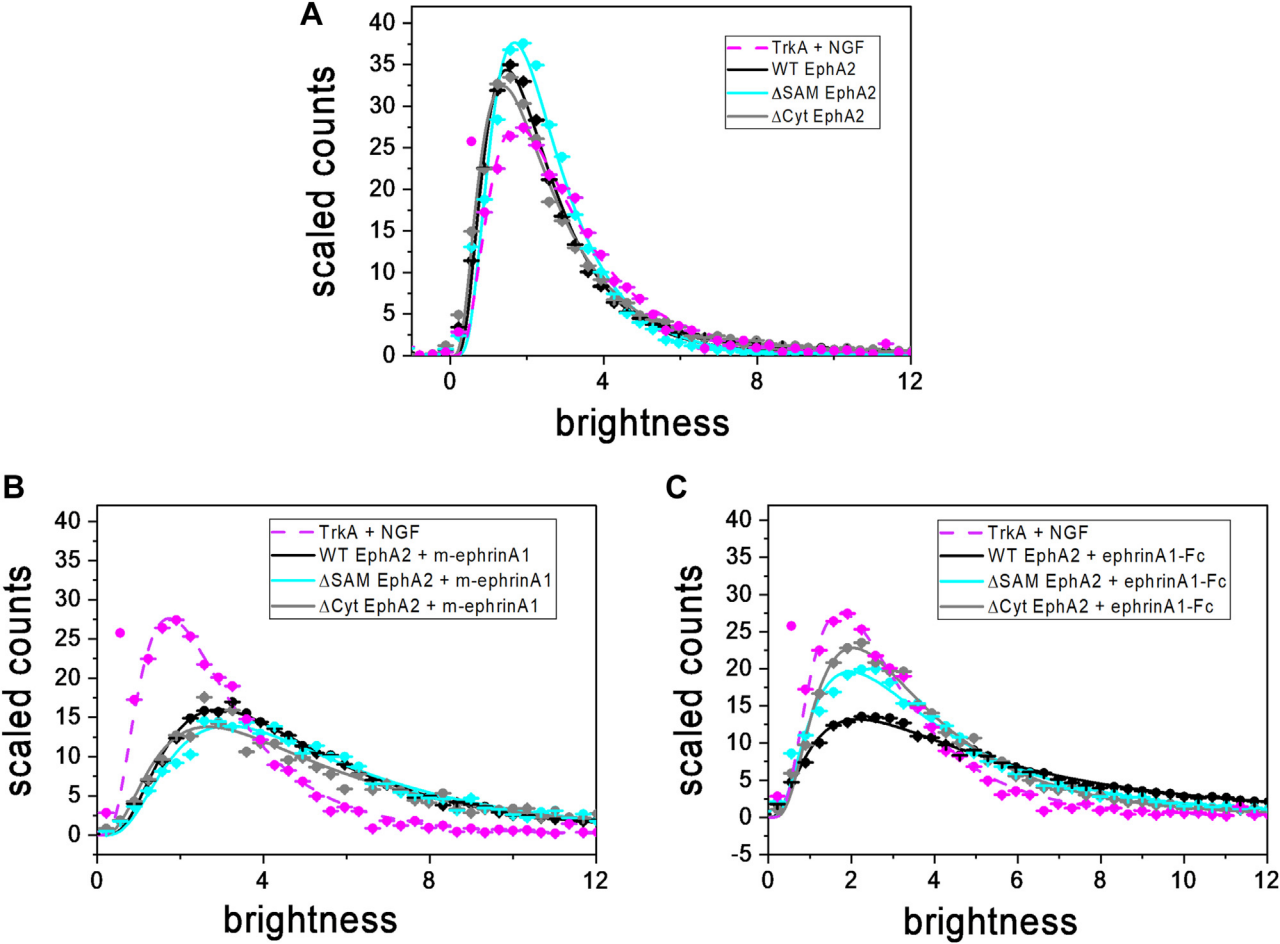
**FIF molecular brightness distributions.** Distributions for EphA2 WT, ΔSAM, and ΔCyt in the absence of ligand (*A*) and in the presence of 200 nM m-ephrinA1 (*B*) and 50 nM ephrinA1-Fc (*C*). The brightness is related to the ratio of the variance and the average of the measured fluorescence intensity, as given by Equation (3). Brightness scales with oligomer size. Molecular brightness distributions were normalized to the area under the curve and fitted with a log-normal distribution function (Equation 4). TrkA + NGF is the dimer control (86). Data for TrkA and EphA2 WT are from Ref. (27). FIF, fluorescence intensity fluctuation; m-ephrinA1, monomeric ephrinA1; NGF, nerve growth factor; SAM, sterile alpha motif.

Previous FIF experiments revealed that EphA2 WT fused at the C terminus to enhanced YFP (eYFP) *via* a flexible (GGS)_5_ linker (EphA2 WT; Fig. 2*A*) can form dimers but no higher oligomers in the absence of ligand (27). However, both m-ephrinA1 and ephrinA1-Fc promote the assembly of EphA2 oligomers larger than dimers (27). Indeed, while the distribution of brightness values for EphA2 WT in the absence of ligand is similar to the distribution of the TrkA + nerve growth factor dimer control (Fig. 3*A*), the distributions are shifted to higher brightnesses by stimulation with m-ephrinA1 and ephrinA1-Fc (Fig. 3, *B* and *C*).

Here, we performed FIF experiments with two truncated mutants, EphA2 ΔSAM and EphA2 ΔCyt, also fused at the C terminus to eYFP *via* a flexible (GGS)_5_ linker. EphA2 ΔSAM lacks the sequence encoding amino acids 902 to 971, which includes the SAM domain (amino acids 904–968) (Fig. 2*A*). EphA2 ΔCyt lacks amino acids 569 to 976, which correspond to the entire EphA2 intracellular region with the exception of the 10 amino acids that immediately follow the transmembrane helix (Fig. 2*A*). Both EphA2 mutants were well expressed and trafficked to the plasma membrane similarly to EphA2 WT, as evident from their localization in plasma membrane–derived vesicles (Fig. 2, *B* and *C*). For FIF experiments, the plasmids were introduced into human embryonic kidney 293T (HEK293T) cells *via* transient transfection, the cells were imaged using a confocal microscope, and the images were analyzed with the FIF software (42). The ligands were used at saturating/near-saturating concentrations (200 nM m-ephrinA1 or 50 nM ephrinA1-Fc; Fig. 1*A*), which are the same concentrations we used in prior work (27, 43).

The calculated molecular brightness values were used to create histograms, which were scaled by integrating the curves and normalizing them so that the areas under the curves are the same (Fig. 3). The normalized molecular brightness distributions were fitted with log-normal functions (Equation 4) to determine the best-fit parameters of the distributions and the mean brightness, as discussed in the *Experimental procedures* section (Table 1).

**Table 1:** Best-fit FIF parameters

Protein	μ	ω	Mean
TrkA + NGF^a^	0.94 ± 0.01	0.62 ± 0.01	3.1 ± 0.1
EphA2 WT^a^	0.74 ± 0.01	0.60 ± 0.01	2.5 ± 0.1
EphA2 ΔSAM	0.81 ± 0.01	0.54 ± 0.01	2.6 ± 0.1
EphA2 ΔCyt	0.76 ± 0.01	0.67 ± 0.01	2.7 ± 0.1
EphA2 WT + m-ephrinA1^a^	1.51 ± 0.01	0.66 ± 0.01	5.6 ± 0.1
EphA2 ΔSAM + m-ephrinA1	1.61 ± 0.01	0.67 ± 0.01	6.2 ± 0.1
EphA2 ΔCyt + m-ephrinA1	1.55 ± 0.01	0.75 ± 0.01	6.3 ± 0.1
EphA2 WT + ephrinA1-Fc^a^	1.57 ± 0.01	0.89 ± 0.01	7.2 ± 0.1
EphA2 ΔSAM + ephrinA1-Fc	1.24 ± 0.01	0.75 ± 0.01	4.6 ± 0.1
EphA2 ΔCyt + ephrinA1-Fc	1.15 ± 0.01	0.67 ± 0.01	4.0 ± 0.1

NGF, nerve growth factor.

Shown are the two best-fit parameters μ and ω (Equation (4)) as well as the means (Equation (5)) of the brightness distributions shown in Figure 3, along with the standard errors. As control, we show data for a known dimer, TrkA + NGF (27, 86).

a Data from (27).

FIF data in the absence of ligand (Fig. 3*A*) show that both EphA2 truncated mutants, similar to EphA2 WT, exist mainly as monomers and dimers, since the distributions of brightness values are slightly shifted to the left compared with the distribution of the dimer control, and the means in Table 1 are lower than the mean for the dimer control. However, in the presence of m-ephrinA1 or ephrinA1-Fc, the mean brightness values for all EphA2 variants are larger than those for the dimer control and EphA2 in the absence of ligand (Fig. 3 and Table 1). Comparison with the dimer control shows that both EphA2 truncated mutants, similar to EphA2 WT, can form oligomers that are larger than dimers when m-ephrinA1 or ephrinA1-Fc is bound (*p* < 0.0001 for each of the comparisons with dimer control). By comparing the EphA2 WT FIF brightness distribution to the distributions of the EphA2 truncated mutants, we found that the effect of the EphA2 intracellular deletions is different in the cases of m-ephrinA1 and ephrinA1-Fc (Fig. 3, *B* and *C* and Tables 1 and 2). In the presence of m-ephrinA1, deletion of either the SAM domain or the entire EphA2 intracellular portion leads to a modest but statistically significant increase in the mean brightness, indicating a modest shift toward higher average oligomer sizes. In the presence of ephrinA1-Fc, deletion of the SAM domain or of the entire EphA2 intracellular region causes a decrease in the mean brightness, indicating the presence of smaller oligomers. This supports the notion that the EphA2 intracellular region contributes in different ways to receptor oligomerization induced by the m-ephrinA1 or ephrinA1-Fc ligands.

**Table 2:** Statistical analysis of the parameters calculated from FIF brightness distributions

Ligand	Comparison	*p* (μ)	*p* (ω)	*p* (mean)
m-ephrinA1	ΔSAM *versus* WT	<0.0001	0.7595	<0.0001
	ΔCyt *versus* WT	0.0138	<0.0001	<0.0001
	ΔCyt *versus* ΔSAM	<0.0001	<0.0001	0.7595
ephrinA1-Fc	ΔSAM *versus* WT	<0.0001	<0.0001	<0.0001
	ΔCyt *versus* WT	<0.0001	<0.0001	<0.0001
	ΔCyt *versus* ΔSAM	<0.0001	<0.0001	<0.0001

Analysis of the values reported in Table 1 was performed using one-way ANOVA, followed by Tukey’s multiple comparisons test.

To further characterize the differences in the arrangement of the EphA2 intracellular regions in oligomers induced by m-ephrinA1 or ephrinA1-Fc, we examined how the intracellular deletions affect the stability of the ligand-bound EphA2 oligomers. Stability is quantified through an effective dissociation constant corresponding to the concentration for which 50% of EphA2 molecules are monomeric and 50% are oligomeric, irrespective of the oligomer size (44). Fully quantified spectral imaging FRET (FSI-FRET) is a variation of FRET that yields oligomerization curves and allows determination of effective dissociation constants for RTKs that self-associate in the plasma membrane (30, 45, 46). We used FSI-FRET to examine the two truncated mutants, EphA2 ΔSAM and EphA2 ΔCyt, fused at the C terminus with either mTurquoise or eYFP (a FRET pair). These experiments were performed in parallel with the recently published characterization of EphA2 transmembrane helix mutants and are therefore compared with the same EphA2 WT data (43). The EphA2 plasmids were introduced into HEK293T cells *via* transient transfection.

For FSI-FRET, the cells were imaged using a spectrally resolved two-photon microscope (47). Images of hundreds of individual cells expressing both mTurquoise- and eYFP-labeled EphA2 were analyzed using Equations (6), (7), (8) to obtain (i) the donor concentrations, (ii) the acceptor concentrations, and (iii) the FRET efficiencies in each cell (45), as described in the *Experimental procedures* section. The use of transient transfection allows us to obtain a wide range of receptor concentrations in the plasma membrane (100 to ∼6000 receptors/μm^2^), as needed for FSI-FRET analyses (45).

**Figure 4:**
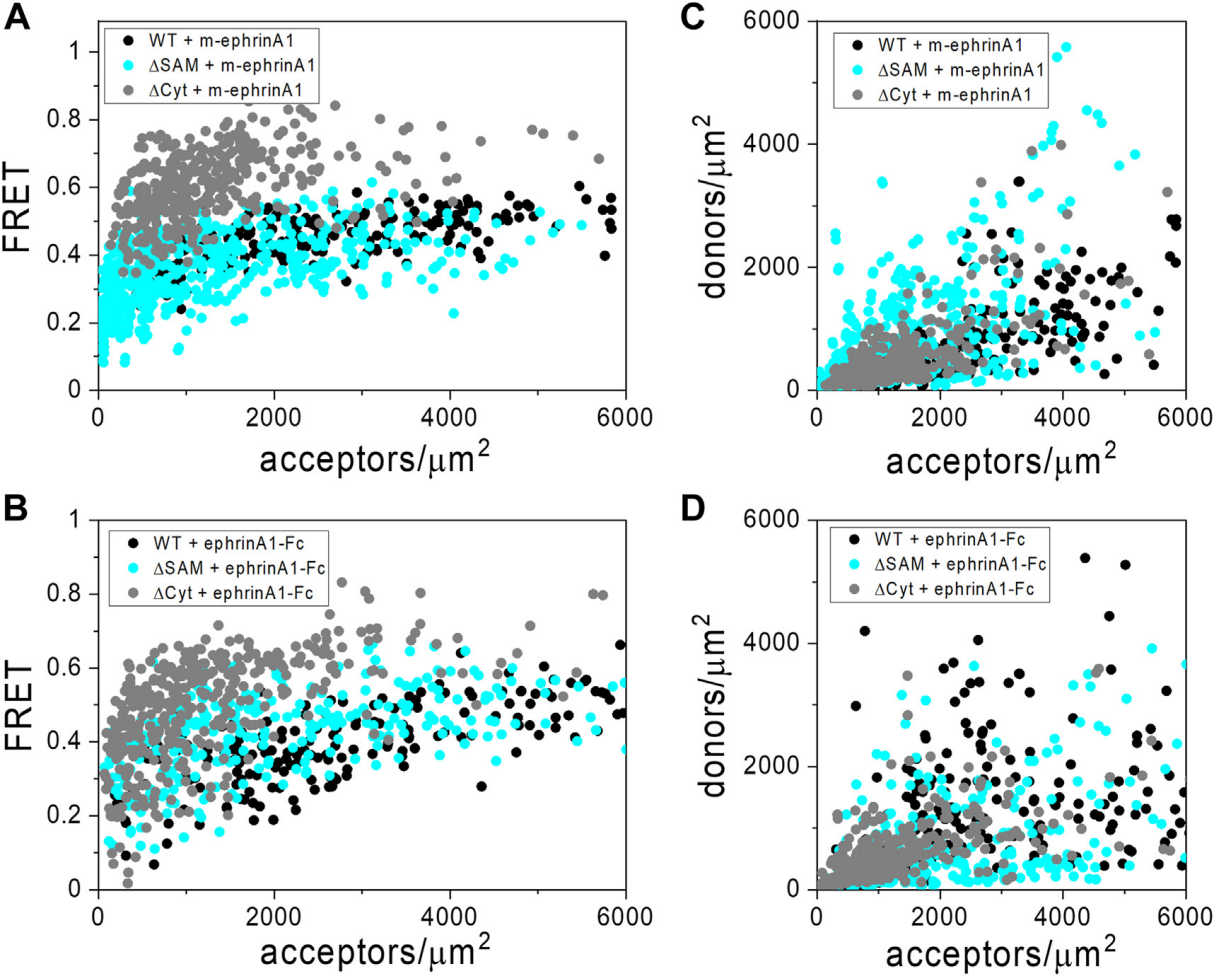
**FRET efficiencies and concentrations from FSI-FRET experiments.** Data for EphA2 WT, ΔSAM, and ΔCyt in the presence of 200 nM m-ephrinA1 (*A*, *C*) and 50 nM ephrinA1-Fc (*B*, *D*). *A* and *B,* FRET efficiency *versus* EphA2-eYFP (acceptor) concentration in individual cells. *C* and *D,* EphA2-mTurquoise (donor) concentration *versus* EphA2-eYFP concentration in individual cells. Data for EphA2 WT are from Ref. (43). FSI, fully quantified spectral imaging; m-ephrinA1, monomeric ephrinA1; SAM, sterile alpha motif.

**Figure 5:**
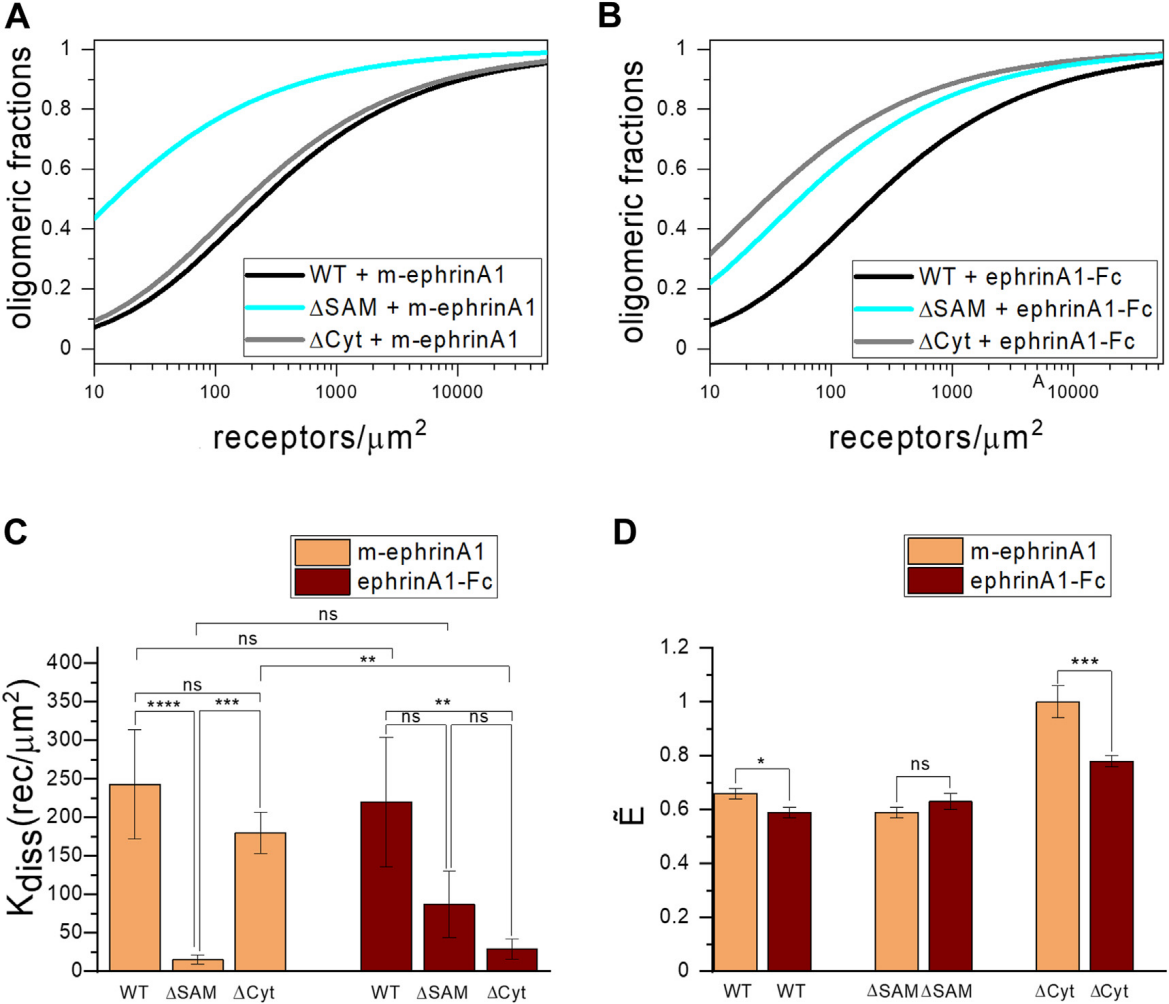
**Comparison of best-fit oligomerization parameters determined in FRET experiments.** Oligomerization curves for EphA2 WT, ΔSAM, and ΔCyt in the presence of 200 nM m-ephrinA1 (*A*) and 50 nM ephrinA1-Fc (*B*). The *solid lines* are fits to all the single-cell data shown in Figure 4. The dissociation constant K_diss_ in Equation (16) is varied to obtain the best fit parameters shown in Table 3. *C,* best-fit dissociation constants measured in the FRET experiments. *D,* best fit intrinsic FRET, Ẽ, values. *C* and *D* are graphical illustrations of the fitted parameters reported in Table 3, Table 4, demonstrating differences in the physical–chemical parameters determined for EphA2 bound to the two ligands, m-ephrinA1 and ephrinA1-Fc. The error bars indicate the standard errors determined from the fits. m-ephrinA1, monomeric ephrinA1; SAM, sterile alpha motif.

The measured FRET efficiencies in the presence of m-ephrinA1 and ephrinA1-Fc increase with acceptor (EphA2-eYFP) concentration for both EphA2 WT and the two EphA2 truncated mutants (Fig. 4, *A* and *B*), consistent with a nonconstitutive association described by the law of mass action. The expression of EphA2-mTurquoise (donor) and EphA2-eYFP (acceptor) varies in different cells, in terms of both donor to acceptor ratio and total EphA2 concentration (Fig. 4, *C* and *D*). Since the measured FRET depends strongly on the relative expression of donors and acceptors (48), as well as the distance between the fluorescent proteins in the different EphA2 oligomers (48), the FRET data for the different EphA2 forms in Figure 4, *A* and *B* cannot be directly compared to inform on the relative strength of EphA2 homointeractions. Instead, we determined the oligomeric fractions and calculated the effective dissociation constants (K_diss_) for EphA2 WT and the two truncated mutants by fitting a model of receptor oligomerization to the FRET data (Equation 16), as described in the *Experimental procedures* section. The results of the fit can be seen in Figure 5, *A* and *B*, which shows the oligomeric fractions plotted *versus* EphA2 concentration for the oligomerized EphA2 WT and mutant forms, and in Table 3, which shows the best-fit values of K_diss_. K_diss_ is determined using Equation (17) as the concentration for which the receptor oligomeric fraction is 50% (44). Lower K_diss_ corresponds to higher oligomer stabilities and *vice versa*.

**Table 3:** Best-fit parameters for the FRET experiments

Ligand	EphA2	K_diss_ (rec/μm^2^)	Ẽ	N
m-ephrinA1	WT^a^	243 ± 71	0.66 ± 0.02	226
	ΔSAM	15 ± 6	0.59 ± 0.02	397
	ΔCyt	180 ± 27	1.00 ± 0.06	389
ephrinA1-Fc	WT^a^	220 ± 84	0.59 ± 0.02	196
	ΔSAM	87 ± 43	0.63 ± 0.03	285
	ΔCyt	29 ± 13	0.78 ± 0.02	391

K_diss_, effective dissociation constant; Ẽ, intrinsic FRET; N, number of individual cells analyzed in each experiment.

a Data from Ref. (43).

Along with K_diss_, the model has a second fitted parameter, intrinsic FRET Ẽ (Equation 16), which depends on the relative positioning and dynamics of the fluorescence proteins in the oligomers (Table 3). The best-fit K_diss_ and Ẽ values for the different EphA2 forms were compared for statistical significance (Fig. 5, *C* and *D* and Tables 4 and 5). When comparing the K_diss_ values, we find that in the case of m-ephrinA1, deletion of the SAM domain decreases the effective dissociation constant and therefore increases the thermodynamic stability of EphA2 oligomers, whereas deletion of the entire intracellular region did not have a statistically significant effect on EphA2 oligomer stability. In the case of ephrinA1-Fc, deletion of the SAM domain did not have a statistically significant effect on EphA2 oligomer stability, whereas deletion of the entire intracellular region increased the stability of EphA2 oligomers. Comparison of the stability of each EphA2 mutant oligomer bound to m-ephrinA1 or ephrinA1-Fc shows statistically significant differences for EphA2 ΔCyt but not for EphA2 WT or EphA2 ΔSAM (Fig. 5, *C* and *D* and Table 5). These findings suggest different overall organizations of the EphA2 intracellular region in oligomers bound to m-ephrinA1 or ephrinA1-Fc. Furthermore, comparison of best-fit Ẽ values between m-ephrinA1 and ephrinA1-Fc shows statistically significant differences only for EphA2 WT and EphA2 ΔCyt (Table 3), confirming structural differences in the EphA2 oligomers bound to m-ephrinA1 or ephrinA1-Fc.

**Table 4:** Statistical analysis of the best-fit dissociation constant K_diss_ determined in the FRET experiments

Ligand	Comparison	*p* (K_diss_)
m-ephrinA1	ΔSAM *versus* WT	<0.0001
	ΔCyt *versus* WT	0.4296
	ΔCyt *versus* ΔSAM	0.0004
ephrinA1-Fc	ΔSAM *versus* WT	0.1121
	ΔCyt *versus* WT	0.0067
	ΔCyt *versus* ΔSAM	0.5515

Analysis of the data reported in Table 3 was performed using one-way ANOVA, followed by Tukey's multiple comparisons test.

**Table 5:** Statistical analysis of the best-fit K_diss_ and **Ẽ** values determined in the FRET experiments comparing the two ligands

EphA2	*p* (K_diss_)	*p* (**Ẽ**)
WT	0.98	0.044
ΔSAM	0.41	0.967
ΔCyt	0.0042	<0.0001

Analysis of the data reported in Table 3 was performed using one-way ANOVA, followed by Tukey's multiple comparisons test.

## Discussion

“Biased signaling” is defined as the ability of different ligands to differentially activate distinct signaling pathways through a common receptor. Recent studies have suggested that RTKs can engage in biased signaling (1, 3, 49, 50, 51, 52, 53, 54, 55, 56, 57). For instance, a proteomics study has shown differences in the tyrosine phosphorylation pattern of the EphB2 receptor and in its downstream signaling network utilization that depend on the nature of different ephrin ligands (58). Differences in the biological responses to different ligands have also been reported for ERBB receptors (59), fibroblast growth factor receptors (52, 60), and TRK receptors (2, 3).

How information about the identity of a ligand bound to the extracellular region of an RTK is transmitted across the plasma membrane to the intracellular region of the RTK, and then to cytoplasmic effector proteins, is a critically important yet unanswered question (35). There are no high-resolution structures of full-length RTKs, because of their large size and the presence of a hydrophobic transmembrane helix, as well as the presence of unstructured linkers between the domains. Our understanding of RTK activation has been shaped, to a large degree, by crystal structures of extracellular domains unbound or in complex with their ligands and of intracellular kinase domains in active or inactive conformations (6, 61, 62). However, such structures provide limited information about the mechanism of signal transduction across the plasma membrane in response to different ligands. There are crystal structures of isolated RTK extracellular domains bound to different activating ligands, which invariably show some structural differences (50, 63, 64). If and how these structural differences affect signaling, however, has been debated (32, 65, 66, 67, 68, 69).

A hypothesis about the mechanism of receptor-biased signaling postulates that structural differences in the extracellular region of an RTK bound to different ligands are propagated along the length of the RTK oligomer to the intracellular region, resulting in differences in the interaction between the intracellular domains. This determines which cytoplasmic tyrosines are most efficiently phosphorylated, leading to the differential activation of downstream effectors. There are many examples that support the view of conformational change propagation along the length of an RTK. Studies on the epidermal growth factor receptor (EGFR) and other ERBB family members, fibroblast growth factor receptors, and vascular endothelial growth factor receptor 2 have suggested that ligand binding leads to a conformational switch in the transmembrane helix, consistent with structural coupling of the extracellular and transmembrane regions (66, 70, 71, 72, 73, 74). Furthermore, alterations in the interaction between the transmembrane helices of HER2/Neu have been shown to affect kinase activity (67), again suggesting that the transmembrane and intracellular regions are structurally coupled.

Yet, there is no direct proof that the intracellular regions can adopt distinct configurations and interact through distinct interfaces when different ligands are bound to the extracellular regions. This scarcity of data is due at least in part to experimental limitations in obtaining structural information about the kinase domains inside the cell when a ligand is bound to the extracellular region. We reasoned that if the intracellular regions of oligomerized RTKs interact through distinct interfaces when different ligands are bound, then the contributions of the intracellular regions to RTK oligomerization will be different.

Some researchers disagree that structural changes in the extracellular region of an RTK that occur in response to the binding of different ligands can be propagated to the intracellular region, because the linkers between the regions are unstructured. For example, it has been argued that a single ligand-bound EGFR conformation can be coupled to multiple kinase domain arrangements (34). Furthermore, a single EGFR kinase domain arrangement can be coupled to two different extracellular states (32), suggesting that the extracellular and intracellular regions of EGFR can change configuration independently of each other. If the juxtamembrane segment between the transmembrane helix and the kinase domain is flexible, the two intracellular regions in the dimer will interact with each other solely based on their physicochemical properties. In this case, the arrangement of the kinase domains would be the same in RTK oligomers bound to different ligands. As a consequence, the contribution of the intracellular regions to RTK oligomerization will be the same when different ligands are bound.

Here, we chose EphA2 as an RTK suitable for investigating these ideas, since we have demonstrated that EphA2 can engage in biased signaling. For EphA2 activated by m-ephrinA1 and ephrinA1-Fc, biased signaling was first demonstrated by determining bias coefficients for Y588 phosphorylation and AKT inhibition (5). The validity of these previous analyses is further supported by the bias plot shown in Figure 1*B*. Thus, here we used EphA2 to investigate whether bias correlates with differential lateral interactions between intracellular regions in RTK oligomers stabilized by different ligands.

We used a combination of mutagenesis and biophysical characterization to probe for differences in EphA2 oligomers for which no high-resolution structures are available. We deleted either the EphA2 SAM domain or the entire EphA2 intracellular region and used FRET and FIF to quantify the role of these regions in EphA2 oligomers induced by the two ligands, m-ephrinA1 and ephrinA1-Fc.

Previous studies have examined the effects of deleting intracellular regions on both EphA2 oligomerization in the plasma membrane and overall EphA2 function. In prior work, we found that deletion of the SAM domain stabilizes EphA2 assemblies in HEK293T cells in the absence of ligand (46). Shi *et al.* (75) deleted either the SAM domain or the kinase and SAM domains, while preserving the juxtamembrane segment, and observed enhanced oligomerization because of the deletions, in the absence of ligand and in the presence of ephrinA1-Fc.

The SAM domain of EphA2 is known to engage with cytoplasmic proteins such as SHIP2, which also possess a SAM domain (76). Thus, the contribution of the SAM domain to EphA2 self-association in different cellular contexts may be different, depending on the abundance of interaction partners that contain a SAM domain. Phosphorylation of the kinase–SAM linker may also affect the interaction of the SAM domain with the kinase domain, affecting EphA2 oligomerization induced by ligand (62). Here, we have directly compared the contribution of different EphA2 intracellular regions to oligomerization induced by the m-ephrinA1 and ephrinA1-Fc ligands in HEK293T cells. By comparing FRET data for EphA2 WT and ΔCyt, we gain insight into the role of the entire intracellular region in the thermodynamic stability of EphA2 oligomers. This is assessed by determining the effective dissociation constant, which denotes the EphA2 concentration for which half of the EphA2 molecules are oligomerized. The presence of the EphA2 intracellular region does not have a statistically significant effect on the dissociation constant in the presence of m-ephrinA1, and thus this region does not significantly contribute to the thermodynamic stability of EphA2 oligomers bound to m-ephrinA1. However, the intracellular region stabilizes EphA2 oligomers bound to ephrinA1-Fc. In addition, the FIF data reporting on the average oligomer size show that the intracellular region slightly inhibits the formation of large EphA2 oligomers in the presence of m-ephrinA1 but notably promotes the association of EphA2 into larger oligomers in the presence of ephrinA1-Fc.

By comparing FRET data for EphA2 WT and ΔSAM oligomers, we gain insight into the role of the SAM domain. This reveals that the SAM domain increases the dissociation constant and thus destabilizes EphA2 oligomers bound to m-ephrinA1, while not having a statistically significant effect on the thermodynamic stability of EphA2 oligomers bound to ephrinA1-Fc. Furthermore, the FIF data show that the SAM domain inhibits the formation of large EphA2 oligomers, thus promoting lower oligomer sizes for EphA2 bound to m-ephrinA1. However, the effects are modest. In contrast, the SAM domain plays a notable role in promoting the formation of large oligomers bound to ephrinA1-Fc.

By comparing the FRET data for EphA2 ΔCyt and ΔSAM, we gain insight into the role of the juxtamembrane segment and kinase domain, which are present only in EphA2 ΔSAM. The region comprising the juxtamembrane segment and kinase domain decreases the dissociation constant, and thus increases the stability, of m-ephrinA1-bound EphA2 oligomers but has no statistically significant effect on the stability of ephrinA1-Fc-bound oligomers. In addition, FIF data show that the juxtamembrane segment and kinase domain do not significantly affect the average EphA2 oligomer size in the presence of m-ephrinA1 but promote the formation of somewhat larger oligomers in the presence of ephrinA1-Fc.

Prior mutagenesis has shown different organization of the EphA2 extracellular regions in oligomers induced by m-ephrinA1 and ephrinA1-Fc (30). Here, we show that the contributions of the intracellular regions to oligomer stability and the average oligomer size are also different in EphA2 oligomers bound to m-ephrinA1 and ephrinA1-Fc. This implies that EphA2 lateral interactions and intracellular organization in the oligomers are different in response to the two ligands. This supports the notion that information about the identity of the bound ligand is transmitted from the extracellular to the intracellular portions of EphA2, along the length of the receptor in the oligomers. In accordance with previous hypotheses (1), the differential interactions in the intracellular portions of the EphA2 oligomers may result in differential phosphorylation of different EphA2 tyrosine residues in the two types of oligomers, which could lead to differential recruitment of effector proteins. This could explain the differential signaling in response to the two ligands. The observed differences could also affect signaling processes that are kinase independent, such as the phosphorylation of Ser/Thr residues located between the kinase and the SAM domain, which leads to noncanonical EphA2 signaling. Yet, the observed correlation does not necessarily imply that a causal link exists between differential EphA2 interactions and EphA2-biased signaling. Additional work with EphA2 and other RTKs with verified biased signaling is needed to establish a possible causation and to reveal the mechanism behind RTK-biased signaling.

As compared with G-protein–coupled receptors, efforts to design ligands inducing RTK-biased signaling are lagging behind. Current RTK inhibitors mainly target the ATP-binding pocket. Often, they are meant to indiscriminately attenuate RTK signaling and have side effects. Future treatments that bias EphA2 signaling toward preferentially inhibiting oncogenic pathways may have utility in the clinic. Here, we find that the differential interactions of intracellular regions in EphA2 oligomers correlate with differential signaling. We can thus hypothesize that EphA2 intracellular interactions might be modulated by molecules that target sites located far from the active site to bias signaling in desirable ways. For example, against cancer, it would be desirable to develop ligands that preferentially inhibit known major oncogenic pathways, such as AKT–mTORC1 and RAS–ERK (77, 78). Alternatively, ligands that bias EphA2 signaling toward promoting receptor endocytosis could be used for delivery of conjugated drugs into EphA2-expressing cells (79, 80, 81) or for depletion of cell-surface EphA2 in order to inhibit parasitic infections (11, 26). Furthermore, ligands that preferentially promote EphA2 degradation could diminish the oncogenic effects of EphA2 S897-mediated noncanonical signaling (11, 12, 82). Better understanding of the mechanisms underlying EphA2-biased signaling will enable the design of ligands that can fine-tune the nature of EphA2 signaling responses as needed.

## Experimental procedures

### Construction of bias plots

Dose–response curves for each ligand were fitted with the Hill equation

(1) $$ResponseA,B=\frac{E_{top}*L^{n_{A,B}}}{{{EC}_{50}}^{n_{A,B}}+L^{n_{A,B}}}$$

where A and B denote the type of response measured, pY588 phosphorylation (A) and AKT inhibition (B). EC_50_ and E_top_ denote the potency and the efficacy of the ligand to induce a response.

The Hill slope *n* was fixed at 2, as this was the lowest n for which fits with R^2^ >0.95 were achieved for the two responses and two ligands.

The bias plot theoretical curves were constructed from the best-fit EC_50_ and E_top_ for each ligand using the following equation (41):

(2) $$Y=\frac{E_{top,B}*{\left(\frac{X*{\left({EC}_{50,A}\right)}^{n_{A}}}{E_{top,A}-X}\right)}^{\frac{n_{B}}{n_{A}}}}{{\left({EC}_{50,B}\right)}^{n_{B}}+{\left(\frac{X*{\left({EC}_{50,A}\right)}^{n_{A}}}{E_{top,A}-X}\right)}^{\frac{n_{B}}{n_{A}}}}$$

The errors were calculated using the so-called “functional approach for multivariable functions” (41), based on the 68% confidence intervals of the fitted EC_50_ and E_top_.

### Plasmids

All EphA2 constructs were in the pcDNA3.1 (+) mammalian expression vector and encoded a C-terminal flexible 15 amino acid (GGS)_5_ linker followed by eYFP or mTurquoise. The EphA2 ΔSAM-eYFP and EphA2 ΔSAM-mTurquoise constructs have been described and used in prior work (30). The EphA2 SAM domain comprises amino acids 904 to 968, and the EphA2 ΔSAM constructs lack the sequence encoding amino acids 902 to 971. The EphA2 ΔCyt-eYFP and EphA2 ΔCyt-mTurquoise constructs lack most of the intracellular domain. They were truncated at amino acid 568 and thus include the transmembrane helix (amino acids 538–558) and a stretch of polar amino acids rich in basic residues: R, K, and H (559–568).

### Cell culture

HEK293T from American Type Culture Collection cells were cultured in Dulbecco's modified Eagle medium, supplemented with 10% fetal bovine serum (ThermoFisher), 1.8 g/l d-glucose, and 1.5 g/l sodium bicarbonate. About 2.0 x 10^4^ cells per dish were seeded in 35-mm glass-bottom collagen-coated dishes (MatTek's Corporation) and kept in an incubator at 37 °C with 5% carbon dioxide. Cells were tested for mycoplasma and were free from mycoplasma contamination.

The production of plasma membrane–derived vesicles followed the protocol described in Ref. (83).

### Transfection

Cells were transfected with 1 to 2 μg of DNA using Lipofectamine 3000 (Invitrogen) according to the manufacturer's recommended protocol. About 12 h after transfection, the cells were rinsed and starved for 12 h in phenol red– and serum-free medium containing 0.1% w/v bovine serum albumin.

### FIF imaging and analysis

The plasma membranes of cells transfected with EphA2-eYFP variants were imaged in a Leica SP8 confocal microscope using a photon counting detector. eYFP was excited using a 488 nm diode laser at 0.1% to avoid photobleaching, at a scanning speed of 20 Hz. About 100 to 150 images were collected for each condition, containing a total of 200 to 300 cells. A region of interest in the plasma membrane of each cell was selected and divided into segments of 15 x 15 pixels (225 pixels). Histograms of intensities were created for each segment, and fitted with a Gaussian function, yielding two parameters: <*I*_*segment*_>, the center of the Gaussian, and *σ*_*segment*_, the width of the Gaussian for each segment.

The molecular brightness of each segment, *ε*_*segment*_, was calculated as:

(3) $$\varepsilon_{segment}=\frac{\sigma_{segment}^{2}}{\left\langle I_{segment}\right\rangle }-1$$

The brightness values from thousands of segments were binned and histogrammed to produce the spectra in Figure 1. These spectra were fitted using Origin Lab with a log-normal function given by:

(4) $$y=\frac{A}{\omega x\sqrt{2\pi }}\;\exp\left(-\frac{{\left(\ln\left(x\right)-\mu \right)}^{2}}{2\omega^{2}}\right)$$

Here, μ is the mean of the respective ln(x) Gaussian distribution and ω is the width of the distribution. The mean of the log-normal distribution is calculated from these two parameters as:

(5) $$mean=\exp\left(\mu +\left(\frac{\omega^{2}}{2}\right)\right)$$

### FRET imaging and analysis

FRET experiments were conducted with the FRET pair of mTurquoise (donor) and eYFP (acceptor) and utilized a two-photon microscope equipped with the OptiMiS True Line Spectral Imaging system (Aurora Spectral Technologies). A Mai Tai laser (Spectra-Physics) was used to generate femtosecond mode–locked pulses. The FSI-FRET method was used to process the spectral images and ultimately produce an association curve (oligomeric fraction *versus* receptor concentration), which yields a dissociation constant as a measure of oligomer stability.

The FSI-FRET method, which has been described in detail previously (30, 45, 46), relies on two sets of controls in each experiment. The first set of controls are soluble mTurquoise and eYFP fluorescent protein solutions of known concentrations. They are used for calibration to determine EphA2-mTurquoise and EphA2-eYFP concentrations in the membrane. The second set of controls are singly transfected cells expressing only EphA2-mTurquoise or only EphA2-eYFP, which are used to unmix the FRET spectra into donor and acceptor components. Each of these controls, as well as cells cotransfected with both EphA2-mTurquoise and EphA2-eYFP, were imaged twice in the fluorescence microscope. The first image was acquired while exciting the donor ($\lambda_{D}=840nm$), and the second image was acquired while exciting the acceptor ($\lambda_{A}=960nm$). Then, the concentrations of donors [*D*] and acceptors [*A*] and the FRET efficiencies in individual cells were determined using the following set of equations derived (45):

(6) $$FRET=1-\frac{F_{\lambda_{1}}^{\text{DA}}}{F_{\lambda_{1}}^{D}}$$

(7) $$\left[D\right]=\frac{F_{\lambda_{1}}^{D}}{i_{D,\lambda_{1}}}=\frac{F_{\lambda_{1}}^{\text{DA}}}{i_{D,\lambda_{1}}}+\frac{Q^{D}}{Q^{A}}\left(F_{\lambda_{1}}^{\text{AD}}-\frac{i_{A,\lambda_{1}}}{i_{A,\lambda_{2}}}F_{\lambda_{2}}^{A}\right)$$

(8) $$\left[A\right]=\frac{F_{\lambda_{2}}^{A}}{i_{A,\lambda_{2}}}=\frac{1}{i_{A},\lambda_{2}}\left(F_{\lambda_{2}}^{\text{AD}}-\frac{i_{D,\lambda_{2}}}{i_{D,\lambda_{1}}}F_{\lambda_{1}}^{AD}\right)\cdot {\left(1-\frac{i_{A,\lambda_{1}}}{i_{A,\lambda_{2}}}\frac{i_{D,\lambda_{2}}}{i_{D,\lambda_{1}}}\right)}^{-1}$$

In these equations, $F_{\lambda_{1,2}}^{D,A}$ is the total fluorescence of the donor or acceptor in the absence of FRET for excitation at $\lambda_{1}$ or $\lambda_{2}$. $F_{\lambda_{1}}^{\text{DA}}$ is the measured fluorescence of the donor in the presence of acceptors, and $F_{\lambda_{2}}^{\text{AD}}$ is the measured fluorescence of the acceptor, which is enhanced because of FRET. $Q^{D}$ and $Q^{A}$ are the quantum yields of the donor and acceptor, respectively. To determine [A] and [D] as two-dimensional concentrations in the membrane, the pixel-level fluorescence intensities for $F^{D}$, $F^{A}$, and $F^{\text{AD}}$ are integrated over a membrane region. The 3D concentrations are converted into 2D concentrations by multiplying the mean integrated fluorescence by the pixel width (45).

As discussed previously (44), comparisons of FRET efficiencies can lead to misleading results, as the total concentrations, and the donor to acceptor ratios, vary from cell to cell. Therefore, to compare the different EphA2 variants, we calculated the effective dissociation constants, which describe the stability of the EphA2 oligomers independently of the oligomer size, n (44). For this calculation, we followed a published protocol (44), which assumes that the behavior of EphA2 is described by the following monomer–oligomer model with an oligomer order (size) n:

(9) m+m…+m⇌oligomer

The dissociation constant for this model, for any n, is defined as (44):

(10) $$K_{D}^{oligomer}=\frac{{\left[m\right]}^{n}}{\left[oligomer\right]}$$

Here, [*m*] is the monomer concentration and [*oligomer*] is the oligomer concentration.

The total concentration of receptors [T] is given by the following equation:

(11) n[oligomer]+[m]=[T]

Combining Equations (10), (11), we obtain

(12) $$\frac{n{\left[m\right]}^{n}}{K_{D}^{oligomer}}+\left[m\right]=\left[T\right]$$

The solution of Equation (12) depends on the oligomer order n, and in the general case, it needs to be solved numerically as a function of [T], $K_{D}^{oligomer}$, and n. We use a root finding MATLAB function to solve for [m], and we take the real positive root to calculate:

(13) $$\left[m\right]=\left[m\right]\left(\left[T\right],K_{D}^{oligomer},n\right)$$

Once *m* is calculated numerically, we calculate the oligomer fraction, that is, the ratio of the concentration of receptors in oligomers and the total concentration.

(14) $$f_{oligomer}=1-f_{monomer}=1-\frac{\left[m\right]}{\left[T\right]}=1-\frac{\left[m\right]\left(\left[T\right],K_{D}^{oligomer},n\right)}{\left[T\right]}$$

The oligomer fraction is related to the FRET efficiency according to (48, 84):

(15) $$FRET=\frac{f_{oligomer}}{x_{D}}\sum_{k=1}^{n-1}\frac{k\left(n-k\right)\tilde{E}}{1+\left(n-k-1\right)\tilde{E}}\left(\begin{array}{c} n\\ k \end{array}\right)x_{D}^{k}x_{A}^{n-k}$$

where $x_{D}\;\&\;x_{A}$ are the fraction of donors and acceptors, and $\tilde{E}$ is the so-called “intrinsic FRET,” which depends on the distance between the fluorophores in the oligomer (44). Combining Equations (14), (15), we arrive at:

(16) $$FRET=\frac{\left[T\right]-\left[m\right]\left(\left[T\right],K_{D}^{oligomer},n\right)}{\left[T\right]x_{D}}\;\sum_{k=1}^{n-1}\frac{k\left(n-k\right)\tilde{E}}{1+\left(n-k-1\right)\tilde{E}}\left(\begin{array}{c} n\\ k \end{array}\right)x_{D}^{k}x_{A}^{n-k}$$

Equation (16) is used to fit the data for any oligomerization model (any given n). $x_{A}$, $x_{D}$, [T], and $E_{oligomer}$ (after correction for proximity FRET (48)) are measured in the experiment. $K_{D}^{oligomer}$ and $\tilde{E}$ are the unknowns that are varied until the model produces the best fit to the FRET data. The fitting was performed using the built-in matlab function “nlinfit.” The errors were calculated with the built-in matlab function “nlparci” where 68% confidence intervals were specified.

The $K_{D}^{oligomer}$ dissociation constant, which has units of (receptors/μm^2^)^*n*-1^, is then used to calculate an effective dissociation constant with units of EphA2 concentration in the membrane according to (44):

(17) $$K_{diss}=\left[T^{*}\right]=2{\left(\frac{K_{D}^{oligomer}}{n}\right)}^{\frac{1}{n-1}}$$

As shown previously, $K_{diss}$ does not depend on the value of *n* used in the fit (44). The physical meaning of $K_{diss}$ is the concentration for which 50% of the receptors are associated into oligomers (and thus active) and 50% are monomeric (and thus inactive).

### Fluorescent protein standards

Soluble monomeric eYFP and mTurquoise fluorescent proteins with an N-terminal 6x His tag were expressed and purified as described (85). The stock solutions were filtered with a 0.2 μm syringe filter. Calibration solutions in the micromolar range were prepared *via* serial dilution. The absorption of eYFP (λ_abs_ = 514 nm) and mTurquoise (λ_abs_ = 434 nm) in the solution standards was measured in a NanoDrop 2000C (Thermo Scientific). Molar absorption coefficients of 83,400 and 30,000 M^-1^cm^-1^ were used for the concentration calculations of eYFP and mTurquoise, respectively.

### Statistical analyses of FRET and FIF data

Best-fit FRET parameters and the FIF means calculated with Equation (4, 5 and 17) were compared using one-way ANOVA, followed by a Tukey’s multiple comparison *post hoc* test. The analysis was performed using Prism 9 (GraphPad Software, Inc). The input in Prism was the best-fit value of a parameter, the standard error, and the number of cells analyzed in the experiments, and the output was a *p* value for each pairwise comparison for two conditions. The results are shown in Tables 2, 4, and 5.

### Western blots

After transfection, HEK293T cells were lysed using radioimmunoprecipitation assay RIPA lysis buffer. Whole-cell lysates were prepared with 4× Laemmli sample buffer with 10% (v/v) β-mercaptoethanol. Lysates were resolved on 7.5% SDS-PAGE gels and transferred to polyvinylidene difluoride membranes (Merck Millipore). Membranes were blocked in 5% bovine serum albumin in Tris-buffered saline with 0.1% Tween-20 for 1 h at room temperature, followed by incubation with primary antibodies at 4 °C overnight. After incubation, membranes were washed four times for 5 min each with Tris-buffered saline with 0.1% Tween-20 and then incubated with horseradish peroxidase–conjugated secondary antibodies for 2 h at room temperature. After a final wash (4 × 5 min), membranes were exposed to SuperSignal West Pico PLUS Chemiluminescent Substrate (Thermo Scientific), and signals were detected using the ChemiDoc XRS + Imaging System (Bio-Rad). For sequential detection, membranes were stripped using Restore Western Blot Stripping Buffer (Thermo Fisher Scientific).

The following primary antibodies were used for detection: goat antihuman EphA2 (catalog no.: AF3035) from R&D Systems, rabbit anti-GFP (catalog no.: 2555) from Cell Signaling Technology, and rabbit anti-β-Actin (catalog no.: 4967). The secondary antibodies conjugated with horseradish peroxidase were donkey anti-goat IgG (H+L) (catalog no.: A15999) from Invitrogen and goat anti-rabbit IgG (H+L) (catalog no.: 111-035-144) from Jackson ImmunoResearch Laboratories.

## Data availability

All data are included in the article.

## Conflict of interest

The authors declare that they have no conflicts of interest with the contents of this article.
